# Gut–lung axis: role of the gut microbiota in non-small cell lung cancer immunotherapy

**DOI:** 10.3389/fonc.2023.1257515

**Published:** 2023-11-22

**Authors:** Huaiyuan Zhang, Ziyuan Xu

**Affiliations:** ^1^ Department of Oncology, Affiliated Hospital of Nanjing University of Chinese Medicine, Jiangsu Province Hospital of Chinese Medicine, Nanjing, Jiangsu, China; ^2^ No. 1 Clinical Medical College, Nanjing University of Chinese Medicine, Nanjing, Jiangsu, China

**Keywords:** gut microbiota, immunotherapy, gut-lung axis, non-small cell lung cancer, fecal microbiota transplant, immune checkpoint inhibitors

## Abstract

Immunotherapy for non-small cell lung cancer (NSCLC) has advanced considerably over the past two decades. In particular, immune checkpoint inhibitors are widely used for treating NSCLC. However, the overall cure and survival rates of patients with NSCLC remain low. Therefore, continuous investigation into complementary treatments is necessary to expand the clinical advantages of immunotherapy to a larger cohort of patients with NSCLC. Recently, the distinctive role of the gut microbiota (GM) in the initiation, progression, and dissemination of cancer has attracted increasing attention. Emerging evidence indicates a close relationship between the gut and lungs, known as the gut–lung axis (GLA). In this review, we aim to provide a comprehensive summary of the current knowledge regarding the connection between the GM and the outcomes of immunotherapy in NSCLC, with particular focus on the recent understanding of GLA. Overall, promising GM-based therapeutic strategies have been observed to improve the effectiveness or reduce the toxicity of immunotherapy in patients with NSCLC, thus advancing the utilization of microbiota precision medicine.

## 1 Introduction

Lung cancer is the leading cause of cancer-related deaths worldwide. In 2022, lung cancer was predicted to cause approximately 350 deaths per day in the United States (1). Non-small cell lung cancer (NSCLC) accounts for approximately 85% of lung cancers (2). Lung adenocarcinoma, squamous cell carcinoma, and large-cell carcinoma are considered the three main NSCLC subtypes (3). In the past two decades, there have been significant advancements in the therapeutic strategies targeting of NSCLC (4, 5).

Immunotherapies currently available for lung cancer include nonspecific immunotherapies, adoptive T-cell immunotherapy, and the use of monoclonal antibodies, oncolytic viruses, or cancer vaccines (3). Immune checkpoint inhibitors (ICIs) are monoclonal antibodies that have been demonstrated to prolong the life of patients with NSCLC; these ICIs have since become a routine treatment for this disease, causing a paradigm shift in the therapeutic management of NSCLC (6–9). Programmed cell death-1 (PD-1), programmed cell death ligand-1 (PD-L1), and monoclonal antibodies against cytotoxic T lymphocyte-associated antigen-4 (CTLA-4) are the most common ICIs currently used for NSCLC; these ICIs restrict T cell effector activity within tissues or downregulate the amplitude of T cell activation (10). ICIs can confer long-term therapeutic effects in some patients with lung cancer. However, only 17–21% of patients with advanced-stage NSCLC respond to ICIs, which is a significantly lower response rate than that seen for melanoma (11). Primary ICI resistance affects 25–44% of patients with NSCLC (12). Furthermore, immune-related adverse events (irAEs) hamper the increased overall survival (OS) of patients treated with ICIs (13). Therefore, studies aimed at enhancing ICI response rates and reducing irAEs are crucial for NSCLC.

To date, numerous combination treatments have been developed to delay or prevent ICI resistance, including modulating the tumor microenvironment (TME), blocking immunological co-inhibitory signals, targeting T-cell priming, and activating T-cells with co-stimulatory roles (14). The gut microbiota (GM) is drawing increasing attention with regard to its ability to shape systemic immune responses and influence ICI efficacy (15). The human body contains up to 4×10^13^ microbial cells, which predominantly consist of bacteria; these bacteria predominantly reside in the gut, accounting for >95% of the total microbial population (16). Additionally, this microbiome contain at least 100 times more genes than our own genome (17). The GM consists of four major phyla (*Bacteroidetes*, *Firmicutes*, *Proteobacteria*, and *Actinobacteria*) and two minor phyla (*Verrucomicrobia* and *Fusobacteria*) (18). Specifically, the GM is considered to be a potent “organ” capable of influencing metabolic, nutritional, physiological, and immunological processes and potentially modulating immunotherapy sensitivity (19).

Nonetheless, the effect of the GM and its metabolites on the efficacy of ICIs in some cancer types such as NSCLC remains poorly understood. In this review, we evaluate the latest information regarding how the GM influences immunotherapy in NSCLC, with an emphasis on the gut–lung axis (GLA). In addition, we summarize the continuing efforts of GM-based manipulation strategies in patients with NSCLC that are receiving immunotherapy. Through this, we aim to provide an effective resource to improve knowledge surrounding the importance of the GM in immunotherapy and NSCLC and provide a foundation for future research.

## 2 Gut microbiota modulates the effectiveness and toxicity of NSCLC immunotherapy

Microorganisms within the gut influence systemic immune responses and affect the efficacy of ICIs through several hallmark mechanisms (12, 15). Multifactorial clinical responses to ICIs can be divided into two categories: tumor-intrinsic factors (mutational status and oncogenic signaling) and tumor-extrinsic factors (microbiota, TME, and metabolic factors) (20). Insufficient production of antitumor T cells, limited function of tumor-specific T cells, and defective establishment of memory T-cells are the three key explanations for ICI therapy failure (21). Mounting evidence has linked the GM with the corresponding patient response to ICI treatment in preclinical and clinical studies (22). The GM augments the intermediate effects of innate immune cells (dendritic cells, macrophages, and natural killer cells), and enhances the antitumor effects of adaptive immune cells (CD8^+^ T cells, CD4^+^ T cells). Additionally, the GM alters TME immunity and host ICI responses (23). The overall effects of GM on the efficacy and toxicity of NSCLC immunotherapy are summarized herein.

### 2.1 Evidence in preclinical mouse models

**Figure 1:**
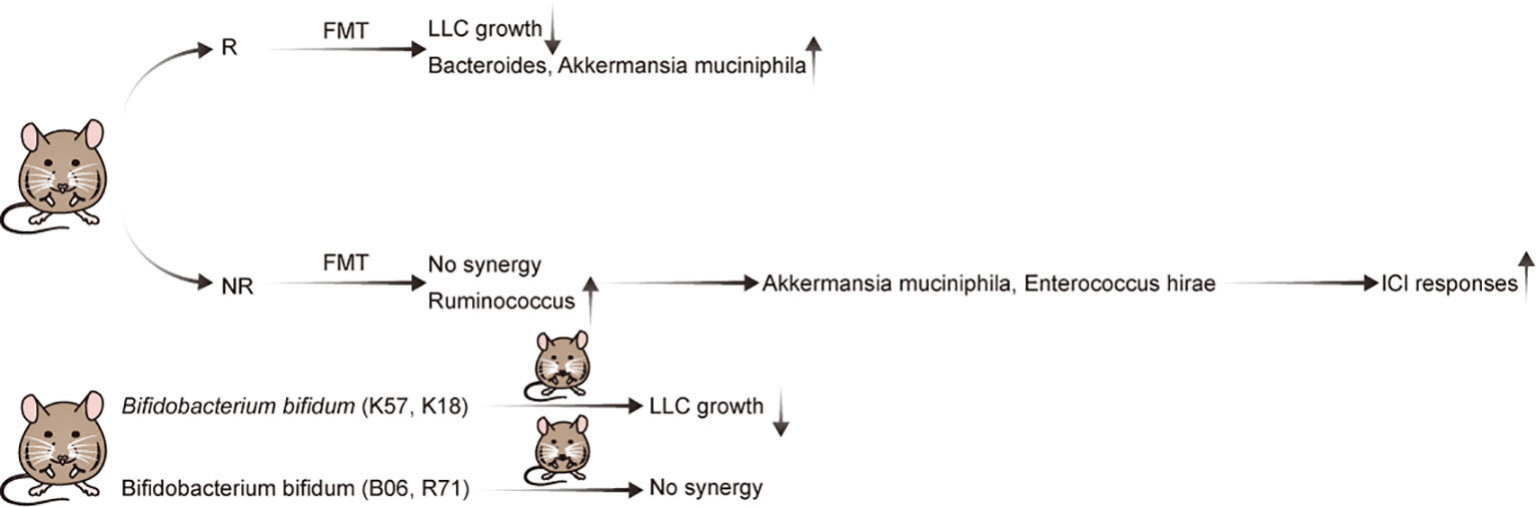
Evidence of the effect of the gut microbiota in preclinical mouse models of NSCLC with immunotherapy.

Preclinical mouse models are important tools for cancer research. The effect of GM on NSCLC immunotherapy was initially illustrated using a mouse model (**Table 1**; **Figure 1**). Individual mice were observed to possess varied responses to NSCLC immunotherapy when fed different GM. Additionally, Newsome et al. transplanted the feces of 65 patients with NSCLC undergoing ICI treatment into germ-free wild-type (GF-WT) mice; this transplantation reduced Lewis lung carcinoma (LLC) growth in these mice. Overall, *Bacteroides* was enriched in the mouse cohort that received transplantation from ICI responders, whereas *Ruminococcus* was considerably enriched in the non-responding mouse group (24). In the same mouse model, Routy et al. demonstrated that the GM is vital for the response to PD-1 blockade. Specifically, they established that mice receiving fecal microbiota transplantation (FMT) from responders exhibited a greater response to anti-PD-1 treatment than mice receiving FMT from non-responsive individuals. In addition, in mice recolonized with fecal microbiota from non-responsive patients with NSCLC, gavage therapy with *Akkermansia muciniphila* alone or in combination with *Enterococcus hirae* alleviated anti-PD1 unresponsiveness (26). Interestingly, they also discovered that the adjuvant impact of *A. muciniphila* on the anti-PD1 response resulted in a corresponding increase in CD4^+^ T cells expressing the chemokine receptor 9(CCR9), which was initiated via the IL-12-dependent signaling pathway (26). Although both studies employed identical LLC mouse models and anti-PD-1 ICIs, differences in the detected bacteria may be attributed to the different experimental conditions.

**Table 1:** Preclinical evidence on the GM-dependent modulation of ICI efficacy in NSCLC.

Year	Tumor model	Treatment	Beneficial bacterial species	Effects	Potential mechanisms	Reference
2022	LLC	Anti-PD-1	*Bacteroides*	Reduced tumor weight	CD8^+^ IFNγ^+^ T cells, CXCR3^+^ CD4^+^ T cells, and tumor-associated macrophages	(24)
2021	LLC	Anti-PD-1	*Bifidobacterium bifidum* (K57 and K18)	Reduced tumor growth	IFN-γ	(25)
2018	LLC	Anti-PD-1	*Akkermansia muciniphila*, *Enterococcus hirae*, and *Alistipes*	Reduced tumor sizes	CCR9^+^CXCR3^+^CD4^+^ T cells via IL-12	(26)

Strain-level differences in the GM can also affect the expression and synthesis of relevant immunomodulatory components in NSCLC and can, thereby, modulate immunotherapy efficacy (25). A key study established that *Bifidobacterium bifidum* is enriched in patients with NSCLC who are responsive to ICI treatment (25). Subsequently, Lee et al. supplemented the GM in syngeneic mouse models with four strains (K57, K18, B06, or R71) of *B. bifidum*. Although all *B. bifidum* strains inhibited LLC growth, only two (K57 and K18) exhibited beneficial synergistic effects with anti-PD-1 therapy (25). Therefore, as not all strains exhibit identical effects, elucidating the underlying mechanisms is crucial for creating live biotherapeutic products (27). Importantly, it was determined that certain strains of *B. bifidum* produce large quantities of peptidoglycan, which activate T cells in NSCLC via peptidoglycan-mediated interferon (IFN)signaling. In mouse models of LLC, this ultimately leads to an enhanced immunotherapy response (25).

Finally, comparable findings have been reported in other preclinical solid tumor models, suggesting that the synergistic effect of GM and ICI therapy may be applicable to ICI-resistant cancer. In preclinical mouse models of melanoma, two studies published in *Science* revealed that PD-1 and CTLA-4 inhibition only suppressed tumor development in mice carrying *Bifidobacterium* and *Bacteroides fragilis* species, respectively (28, 29). Further, Griffin et al. determined that *Enterococcus* spp. with unique NlpC/p60 peptidoglycan hydrolase activity may produce NOD2-active muropeptides; these muropeptides could then alter the effectiveness of checkpoint blockade immunotherapy *in vivo* (30). In mouse models of colorectal cancer, Mager et al. revealed that bacterial inosine is associated with the GM-dependent response to ICIs (31). FMT from responsive and non-responsive patients into mice with human epidermal growth factor (HER2)-positive breast cancer mimicked the responses to ICIs observed in animal models of breast cancer (32). In renal cell carcinoma (RCC) tumor-bearing mice, oral gavage with feces from ICI-responding patients with RCC reversed the initial resistance of these mice to ICI treatment (33). In NSCLC, the relevant research is still blank. However, in melanoma, gut dysbiosis attenuated the anti-tumour efficacy of Tim-3 blockade in mice (34). In colon adenocarcinoma tumor-bearing mice, the immunotoxicity of anti-CD137 immunotherapies is dependent on GM (35).

These preclinical studies have successfully demonstrated that GM composition has a clear impact on checkpoint blockade effectiveness, and alteration of the GM can enhance response to ICI treatment. Nevertheless, most preclinical investigations that demonstrated the influence of GM on ICI response have been limited to melanoma and colorectal cancer. Therefore, NSCLC should receive more attention in future pre-clinical experiments into the effect of the GM on ICI response.

### 2.2 Evidence in patients with NSCLC

The human GM does not necessarily colonize mice in the same manner as it colonizes humans. Furthermore, the mucosal and immunological responses of mice to introduced commensals are not comparable to those in humans (36). Consequently, various research groups have sought to define the function of the GM in NSCLC immunotherapy outcomes to better understand the difference in responders (Rs) and non-responders (NRs) (**Tables 2**, **3**). Overall, 16S rDNA sequencing and metagenomic shotgun sequencing (MSS) were used to analyze the composition of GM in these studies.

**Table 2:** Clinical evidence on the GM-dependent modulation of ICI efficacy in NSCLC.

Study year	Sample size	Treatment	Effects	GM related to the response	Potential interventions and biological effects	Reference
2023	47	Anti-PD-(L)1	longer PFS	*Bacteroidaaceae*, *Barnesiellaceae*, *and Tannerellaceae*	Not discussed	(37)
2023	113	Anti-PD-(L)1	longer PFS and OS	*Anaerostipes* and *Eubacterium ventriosum*	Systemic inflammatory marker-derived neutrophil-to-lymphocyte ratio and lung immune prognostic index	(38)
2022	338	Anti-PD-1	Higher ORR and longer OS	*A. muciniphila*	T_H_1 cells	(39)
2022	16	Anti-PD-1	Improved response and survival rates	*Bacteroides vulgatus*, *Parabacteroides distasonis*, *Bacterium LF-3*, and *Sutterella wadsworthensis HGA0223*	T cells and effector T cells	(40)
2021	75	Anti-PD-1	Improved clinical response	*Alistipes, Anaerostipes*, *Desulfovibrio*, *Faecalibacterium*, and *Bifidobacterium*	Increased antigen presentation and improved effector T cell function	(22)
2021	16	Anti-PD-1	Limited the growth of target lesions	*Escherichia-Shigella*, *Akkermansia*, and *Olsenella*	IL-12 and IFN-γ	(41)
2020	70	Anti-PD-(L)1	Higher ORR and longer PFS	Ruminococcaceae UCG 13 and *Agathobacter*	CD4^+^ and CD8^+^ T cells	(15)
2020	63	Anti-PD-(L)1	Longer PFS	*Parabacteroides* and *Methanobrevibacter*	Not discussed	(42)
2019	37	Anti-PD-1	Longer PFS	*Alistipes putredinis*, *Bifidobacterium longum*, and *Prevotella copri*	Memory CD8^+^ T cells and natural killer cells	(43)
2018	60	Anti-PD-1	Longer PFS	*A. muciniphila*	CD8^+^ T_C_1 T cells	(26)

**ORR,** overall response rate; **OS,** overall survival; **PFS,** progression-free survival.

**Table 3:** Ongoing clinical trials on the association between the intestinal microbiome and immunotherapy in NSCLC (www.clinicaltrials.gov).

NCT#	Age	Sample size	Study description	Location	Investigator	Study type	Length
NCT05037825	18+	800	How microbiota and their byproducts affect cancer immunotherapy	United States	Diane Drobny	Observational	11/21–09/28
NCT04804137	18+	80	The GM tract of patients receiving immunotherapy	France	Marion Ferreira	Observational	05/21–03/25
NCT04063501	18–100	80	Whether microbiota signatures can predict patient response	United States	Leopoldo N Segal	Observational	09/20–09/24
NCT04682327	18-75	50	To analyze the composition and diversity of gut microbiota in patients with advanced or metastatic NSCLC, and to explore the relationship between gut microbiota and response to anti-PD-1/PD-L1 therapy	China	Chen Qi	Observational	08/21-12/22
NCT04189679	18+	60	Identifying predictive metabolic profiles of immune checkpoint inhibitor response in non-small cell lung cancer	France	La Tronche	Observational	01/20-12/23
NCT04136470	18+	130	Detect microbiome differences between cancer patients who respond to immunotherapy and those who do not	Poland	Martyna Balawejder	Observational	04/19-03/21

**Figure 2:**
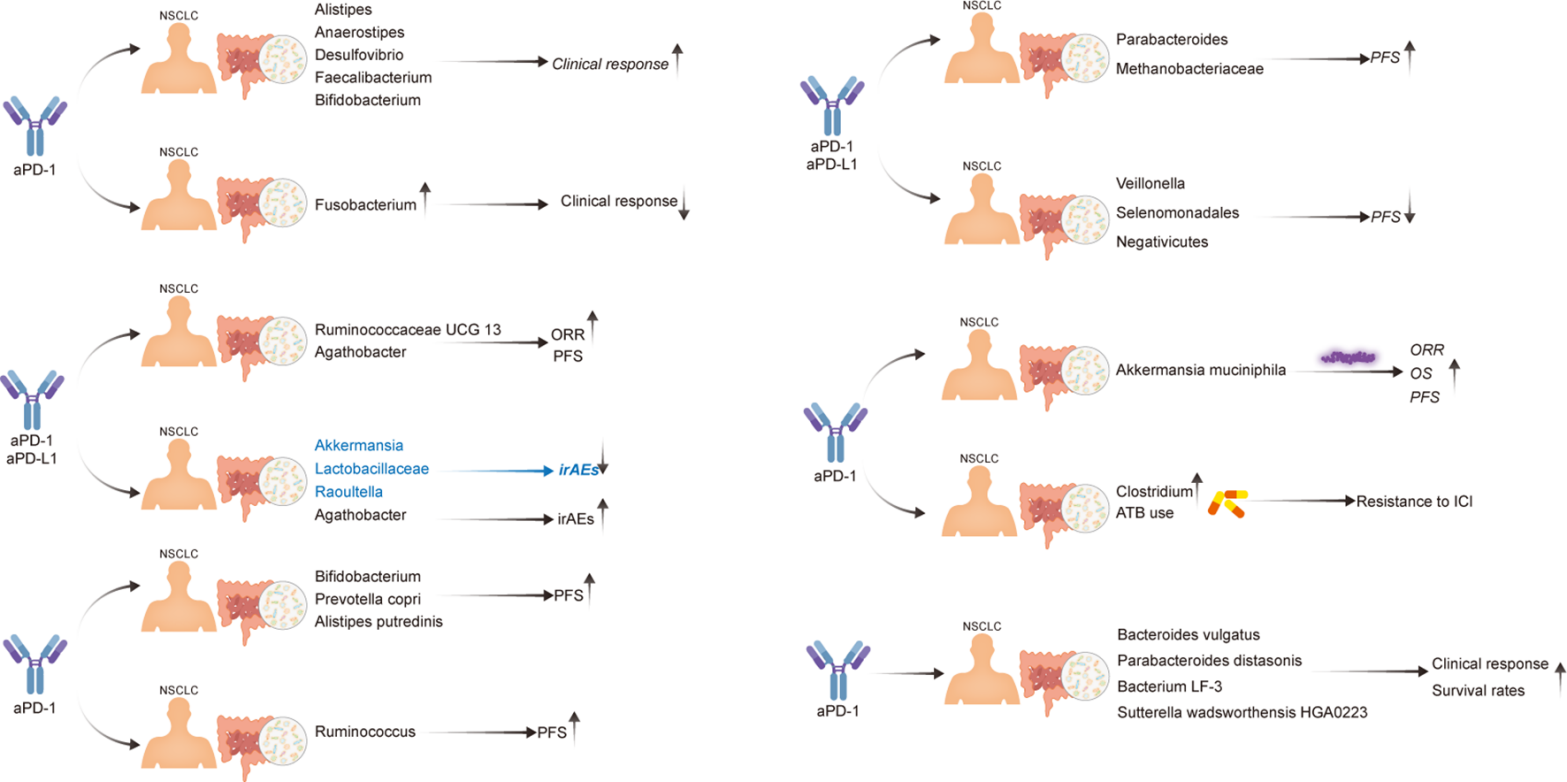
Evidence of the effect of the gut microbiota in patients with NSCLC undergoing immunotherapy.

First, Routy et al. established that *A. muciniphila* is overrepresented in the stool samples of patients with NSCLC who benefit from PD-1 treatment. Specifically, they reported an enrichment of *A. muciniphila* in R *vs*. NR, as well as a relative underrepresentation of *Bifidobacterium adolescentis* and *Parabacteroides distasonis* in those that responded to PD-1 treatment. Antibiotic (ATB) use has also been observed to have a deleterious influence on the immune checkpoint blockade response (26). Four years later, the same research group used microbiome profiling to prospectively evaluate the benefits of fecal *A. muciniphila* in a large cohort of patients with advanced NSCLC (n = 338) who were treated with first- or second-line ICIs. In this study, ATB usage and the *Clostridium* genus were linked to ICI resistance (39). Additionally, Martini et al. provided clinical evidence that two butyrate-producing GM (*Agathobacter* M104/1 and *Blautia* SR1/5) may improve immune responses in patients with metastatic and chemorefractory NSCLC (44). Jin et al. also reported that memory T cell and natural killer cell signatures in peripheral blood were higher in patients with advanced NSCLC with high gut bacterial diversity (43). Therefore, patients with NSCLC with high microbiome diversity were determined to exhibit a longer progression-free survival (PFS) than those with a low-diversity microbiome (43). Five other studies reported similar results; the various GM compositions associated with improved ICI responses in these studies are listed in **Table 2**. Overall, there are substantial variations between the diversity and composition of GM in the R and NR groups in patients with NSCLC. GM profiling in patients with NSCLC revealed that a higher gut bacterial diversity and an increased abundance of specific bacteria, such as *A. muciniphila*, *Bacteroides vulgatus*, *P. distasonis*, *bacterium LF-3*, *Sutterella wadsworthensis HGA0223*, *Agathobacter*, *Blautia*, *Alistipes*, *Anaerostipes*, *Desulfovibrio*, *Faecalibacterium*, *Bifidobacterium*, *Escherichia-Shigella*, *Olsenella*, Ruminococcaceae UCG 13, *Methanobrevibacter*, *Alistipes putredinis*, and *Prevotella copri*, were associated with a stronger T-cell dependent antitumor response, resulting in favorable ICI clinical outcomes in multiple independent trials. Li et al. analyzed the gut microbiota to explore potential causal associations with various subtypes of lung cancer using mendelian randomization. They identified 10 groups associated with lung adenocarcinoma and 9 groups associated with squamous cell lung cancer. Notably, a significant causal relationship between Peptococcaceae and lung adenocarcinoma was observed, as reported in the literature (45). However, the gastrointestinal microbiome does not always play a beneficial role in NSCLC immunotherapy. According to a retrospective investigation of two independent cohorts of patients with NSCLC, *Helicobacter pylori* has been associated with an evident decrease in the PFS of patients with NSCLC undergoing anti-PD-1 therapy (46) (**Figure 2**). Studies have shown that gut microbiota plays an important role in the response of NSCLC patients to anti-PD-1 therapy, and that gut microbiota diversity is significantly increased in patients who respond to anti-PD-1 therapy (47). A prospective study of 63 patients with advanced NSCLC taking anti-PD-1 therapy found that β diversity in gut microbiota at baseline was significantly increased in patients with progression-free survival (PFS)≥ 6 months compared with patients with PFS<6 months; The LEfSe analysis suggested that the most significantly associated groups in patients with PFS≥6 months were *Paracterium* (LDA score=3.8) and *Methanobrevibacterium* (LDA score=3.4), whereas the PFS<6 months group was rich in *Veillonella*, *Seleniomonas*, and *Negativicutes*. Further analysis showed that *Paracobacterium* and *Methanobrevibacterium* species in NSCLC patients enhance their responsiveness to Immune checkpoint inhibitors (42). The results reported in a study showed that in NSCLC receiving first-or second-line immunotherapy to detect its pre-treatment gut microbiota, *Akkermansia muciniphila* (AKK+) patients were found to have a higher immunotherapy response rate than AKK-patients (28% *VS* 18%), among which AKK+ patients, the response rate of first-line immunotherapy was as high as 41%. For survival, overall survival(OS) was longer in the AKK+ group than in the AKK-group (18.8 months *vs* 15.4 months) (39).

In summary, experimental and clinical evidence has demonstrated that GM plays a key role in modifying the effectiveness of immunotherapy in NSCLC. However, these studies have several limitations that should be addressed. For example, most clinical trials have been undertaken on a relatively small number of patients with NSCLC; additionally, current research seems to lack consensus on what GM compositions are advantageous or antagonistic to ICI treatment of NSCLC. Accordingly, several clinical studies are being conducted to address these issues (**Table 3**). Further, the cause-and-effect relationship between the GM and immunotherapy in NSCLC remains unclear. Therefore, further research is required to determine whether the GM modulates immunotherapy in patients with NSCLC or whether immunotherapy alters the GM of patients with NSCLC. Furthermore, the correlations between B cell responses and the GM are yet to be thoroughly investigated in the context of NSCLC immunotherapy.

### 2.3 Microbiota-derived metabolites and immune-related adverse events

Although ICIs are the first-line therapy for NSCLC, several irAEs have been reported. According to a multicenter cohort study, 623 patients with NSCLC treated with PD-(L)1 monotherapy developed multisystem irAEs, the most prevalent of which were pneumonitis thyroiditis, hepatitis thyroiditis, dermatitis thyroiditis, and dermatitis pneumonitis (48). Anti-PD-(L)1-induced checkpoint inhibitor pneumonitis is more common in patients with NSCLC than in those with other malignancies. Disordered T-cell subsets, elevated preexisting and emerging autoantibodies, and imbalanced inflammatory cytokines are three pathways that may explain this PD-(L)1 inhibitor–mediated pulmonary damage (49). Additionally, according to Tang et al., the most frequently observed irAE is checkpoint inhibitor colitis (50).

However, the treatment of irAEs remains a key source of concern in NSCLC immunotherapy. In most cases of irAE, immunotherapy may be withheld or stopped completely, depending on the severity of toxicity (51). Several studies considering the putative link between GM and irAEs in NSCLC have been conducted. Hakozaki et al. obtained pre-ICI fecal samples from 70 Japanese patients with advanced NSCLC who were then treated with ICIs. Ultimately, they determined that *Akkermansia*, *Lactobacillaceae*, and *Raoultella* were associated with milder irAEs, whereas *Agathobacter* was associated with more severe irAEs (15). Another study in which 37 patients were enrolled indicated that the GM of patients who did not experience irAEs was relatively enriched in *Bifidobacterium* and *Desulfovibrio* spp (52). *Bifidobacterium*, *Faecalibacterium*, and *Agathobacter* were also determined to be less abundant in patients with irAEs (53).

Zeng et al. observed notable alterations in butyrate-producing bacteria within a cohort of patients experiencing secondary resistance (SR) to ICIs. There was a downward trend in these bacteria levels upon the onset of secondary resistance. Notably, Bacteroides played a significant role in the variance between the baseline and the occurrence of irAEs. The abundance of Bacteroides declined after the onset of irAEs and subsequently returned to levels comparable to the baseline after remission (54).

Furthermore, a multicenter non-interventional trial (NCT04107168), involving up to 40 UK sites, is currently underway. To examine mechanisms underlying irAEs, longitudinal stool samples were collected prior to NSCLC immunotherapy and following 6 weeks, 3 months, 6 months, and 12 months of treatment. A clear association between GM composition and ICI toxicity was one of the initial results established in this clinical trial (55).

Additionally, the GM has been reported to be associated with irAEs in other cancers. For example, Andrews et al. established that *Bacteroides intestinalis* mediates immune-related intestinal damage via IL-1β in melanoma (56). The link between irAEs and several *Lachnospiraceae* species in melanoma has also been verified by McCulloch et al. (57). Further, Mao et al. determined that a higher GM diversity and relative abundance of the *Firmicutes* phylum may be protective factors against irAEs in patients with hepatobiliary cancer (58). Finally, compared with the post-treatment microbiota of immune-related acute pancreatitis, a low *Bacteroidetes/Firmicutes* ratio, a low relative abundance of *Alistipes* and *Bacteroides*, and a high *Lachnospiraceae* abundance were observed in the baseline microbiota of individuals with pancreatic cancer (59).

## 3 Gut–lung axis

Gut microbiota not only regulates the immune response of gastrointestinal tract, but also influences the health and disease of distal organs such as respiratory system through intestinal microecology. The relationship and interaction between the gut and the lung is called Gut-lung axis (60).

Although the gut and the lungs are physically separate and possess substantially different functions, they share several similarities. First, they share a common embryonic origin and structural similarities (61, 62). Specifically, both organs are derived from the endoderm and consist of columnar epithelial cells with projections of microvilli (gut) or cilia (lungs). Both organs also secrete mucus through goblet cells (63). Additionally, both the gut and the lungs possess an enormous surface area exposed to the external environment; hence, they both serve as the first line of defense against invading foreign pathogens and play important roles in innate and adaptive immunity (64). The GLA hypothesis arises from the understanding that intestinal and respiratory disorders share pathological abnormalities; further, alterations in the gut microenvironment may influence the pathology of various lung disorders (65, 66).

Considerable evidence has indicated that dynamic crosstalk between the gut and the lungs establishes the GLA. Additionally, bronchi content aspiration can deliver up to 10^11^ live bacteria to the intestines each day. Further, gut-derived harmful substances can enter the lung circulation via intestinal lymphatics (67). Huang et al. discovered a unique cell type (group 2 innate lymphoid cells) that resides in the gut but can migrate to the lungs via sphingosine-1-phosphate receptors and can, ultimately, contribute to host defense (68). Moreover, Ruane et al. established that lung dendritic cells, such as CD103(+) mesenteric lymph node (MLN) dendritic cells, stimulate protective T lymphocyte migration to the gastrointestinal tract (69). Further, microbiota administered into the nasal cavity of mice can be observed in the gut following a short incubation period (70).

**Figure 3:**
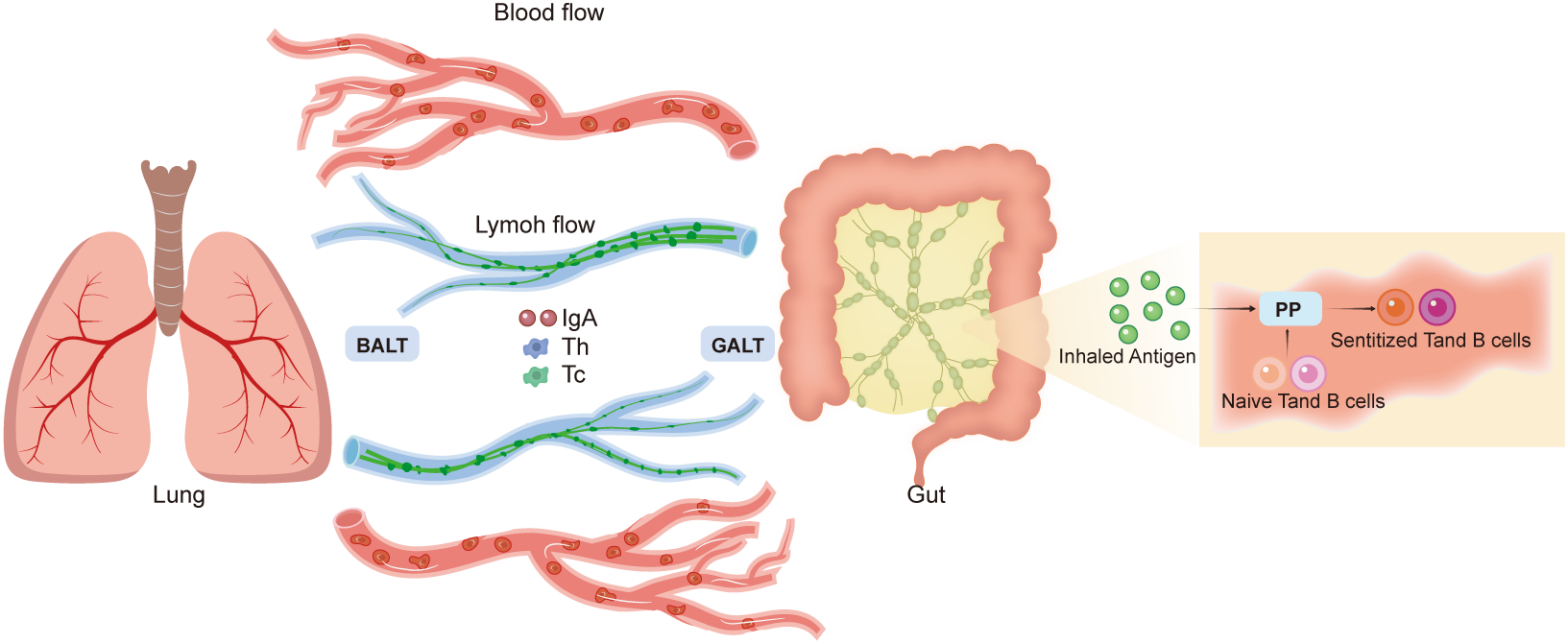
Gut–lung axis. The mechanisms of gut–lung communication are unclear. The common mucosal immune system theory suggests that Peyer’s patches capture inhaled antigens and induce naive T and B cells. These sensitized T and B lymphocytes travel to the mesenteric lymph nodes and bloodstream, which distribute these lymphocytes to effector sites, such as the gut-associated lymphoid tissue (GALT) and bronchus-associated lymphoid tissue (BALT). GALT and BALT play immunological roles and can impact systemic responses. According to this system, circulation of the lymphatic fluid and blood can contribute to gut–lung interactions.

However, the mechanisms mediating the communication between the gut and lungs remain unclear. An idea, named the common mucosal immune system, was proposed in 1978 (71). This system operates according to the following scheme: Peyer’s patches (PP) capture inhaled antigens and transport them to antigen-presenting cells (APCs); naïve T and B cells are induced in the PPs; after sensitization, T and B lymphocytes travel to the MLNs and the thoracic duct to reach the bloodstream; subsequently, these sensitized T and B cells are distributed to several effector sites, including the gut-associated lymphoid tissue (GALT) and bronchus-associated lymphoid tissue (BALT) (67). The key immunological roles of GALT and BALT include IgA synthesis and secretion at mucosal surfaces, alongside establishing helper and cytotoxic T cell responses (72, 73). Local immune responses to GALT and BALT can affect systemic immunological responses; nonetheless, these immune responses can also operate independent of the systemic site (65). Thus, according to the common mucosal immune system, the circulation of lymphatic fluid and blood contributes to the interactions observed between the gut and the lungs (**Figure 3**).

The bidirectional relationship between the gut and the lungs is the foundation for understanding the role of the GM in NSCLC immunotherapy. Different GM can act on the epithelial barrier to activate innate and cognate immune responses against NSCLC antigens; this activation occurs through a range of mechanisms, including endoplasmic reticulum stress, intestinal crypt apoptosis, inflammasome activation, Toll-like receptor signaling, nucleotide-binding oligomerization domain activation, and chemokine receptor signaling (74). In addition to the mucosal immune system, three other mechanisms have been postulated to explain the effects of the GM on lung immunity, which may be associated with NSCLC immunotherapy response rate. First, cytokines and growth factors generated by the gut mucosa in response to the GM may enter the systemic circulation and affect other mucosal tissues. Second, microbial-associated molecular patterns can be absorbed and transported to the lungs, where they can regulate immune responses. Third, metabolites ingested by the GM have been linked to a phenomenon known as “metabolic reprogramming,” which involves the manipulation of mucosal immunity (75); for example, the concentration of circulating short-chain fatty acids (SCFAs) in the intestine affects IL-6 and IL-8 levels at the NSCLC tumor site (76).

Through the down-regulation of mucosal addressin cell adhesion molecule 1 (MAdCAM-1) in the ileum, post-antibiotic (ABX) gut recolonization by Enterocloster species facilitated the migration of enterotropic α4β7CD4 regulatory T 17 cells towards the tumor. Conversely, fecal microbiota transplantation (FMT) or neutralization of interleukin-17A prevented the ABX-induced immunosuppression. Notably, in distinct cohorts of lung cancer patients, reduced serum levels of soluble MAdCAM-1 were associated with an adverse prognosis. This underscores the significance of the MAdCAM-1-α4β7 axis as a modifiable gut immune checkpoint in the context of cancer immunosurveillance (77, 78).

## 4 Strategies to manipulate the GM for NSCLC immunotherapy

There are a number of potential approaches to target regulation of the gut microbiota to enhance NSCLC immunotherapy response rates, including FMT, ATBs, prebiotics, probiotics, postbiotics, synbiotics, bacteriophages, and diet. These approaches can alter the composition and function of the gut microbiota and can be used to promote gut microbiota balance and function in NSCLC immunotherapy.

### 4.1 Fecal microbiota transplantation

FMT involves the transfer of GM from a known donor to a recipient via the upper or lower gastrointestinal route to restore microbial diversity within the intestine (79). In 1958, Eiseman published the first report on the use of FMT for the treatment of severe pseudomembranous enterocolitis (80). Despite being a well-established therapy for recurrent *Clostridium difficile* infections, FMT is now being explored in the context of cancer treatment (81).

FMT is a potential therapy for various types of cancer and cancer treatment–associated complications. In preclinical mouse models, Shaikh et al. utilized FMTs from two NSCLC patients who had contrasting responses to ICIs (R and NR); overall, R-FMT mice exhibited superior antitumor responses when paired with ICI treatment compared to that of NR-FMT mice (11). Baruch et al. and Davar et al. reported the first-in-human clinical trials conducted to determine whether FMT can influence the response of patients with metastatic melanoma to anti–PD-1 immunotherapy. Both trials demonstrated signs of therapeutic benefit in some treated patients, including a higher abundance of taxa associated with an anti–PD-1 response and enhanced CD8^+^ T cell activation (82, 83). Several clinical trials of NSCLC are currently being conducted to evaluate whether combining FMT with immunotherapy can enhance the outcomes of patients with NSCLC (**Table 4**).

**Table 4:** Ongoing clinical trials on the effects of FMT on NSCLC outcomes (www.clinicaltrials.gov).

NCT#	Age	Sample size	Study type	Study description	Location	Investigator	Status	Phase	Length
NCT04951583	18+	70	Interventional	Anti-tumor activity of FMT in combination with ICI therapy	Canada	Bertrand Routy	Recruiting	2	11/21–09/25
NCT04521075	18+	42	Interventional	Safety and efficacy of FMT in combination with nivolumab in patients with metastatic or inoperable NSCLC	Israel	Guy Ben-Betzalel	Recruiting	1 and 2	11/20–07/23
NCT05669846	18+	26	Interventional	Whether FMT improves the body’s ability to fight cancer in patients with relapsed/refractory PD-L1-positive NSCLC	United States	Amy Rose	Not yet recruiting	2	07/23–07/28
NCT05502913	18+	80	Interventional	Safety and efficacy of FMT in altering ICI response in patients with metastatic NSCLC	Israel	Ismaell Massalha	Not yet recruiting	2	09/22–06/25
NCT03819296	18+	800	Interventional	The role of the gut microbiome and effectiveness of FMT on patients with cancer (including NSCLC)	United States	Yinghong Wang	Recruiting	1 and 2	02/21–01/23
NCT05008861	18-75	20	Interventional	To evaluate the safety of FMT in the treatment of advanced NSCLC, and to analyze the effect of FMT on the intestinal flora and immunophenotype of patients	China	Chen Qi	Recruiting	1	09/21- 12/22
NCT05286294	18+	20	Interventional	Modulating patients’ gut microbiome by FMT addresses cancer for which immunotherapy failed	Norway	Jon Amund Kyte	Recruiting	2	06/22-12/34

Despite the promising results of FMT in patients treated with ICIs, concerns remain regarding its long-term safety. According to a study published in *Lancet*, 32 (78%) of the 41 individuals receiving FMT treatment experienced adverse events, the majority of which were self-limiting gastrointestinal problems (84). Nonetheless, a randomized, placebo-controlled trial that evaluated the safety of FMT for active peripheral psoriatic arthritis reported no serious adverse events in patients treated with FMT (85). According to the European consensus on FMT in clinical practice, FMT shows an excellent short-term safety profile, with only a few mild adverse events observed. However, long-term safety data are limited. Therefore, theoretically, FMT may transfer potentially dangerous microbial features as well. In conclusion, given that FMT is a highly effective treatment for NSCLC patients, the benefit-to-risk ratio should be considered for each individual (86).

### 4.2 Antibiotics and proton pump inhibitors

ATBs not only kill pathogenic bacteria but also alter the diversity and composition of the GM, thereby disrupting gut homeostasis (87–89). In the context of NSCLC, ATBs appear to be associated with a reduction in ICI therapeutic impact (**Table 5**). Specifically, PFS duration was considerably reduced in patients with NSCLC who were treated with ATBs within 2 months of their initial ICI therapy than in those who had not received ATB treatment within this timeframe (96). Furthermore, the use of ATBs before ICI therapy has been demonstrated to reduce GM diversity, and the presence of Ruminococcaceae in patients with advanced NSCLC resulted in unfavorable outcomes (15). The effectiveness of cancer therapies has also been revealed to be impaired in ATB-treated and GF mice (97).

**Table 5:** Clinical trials on the effects of ATBs on ICI outcomes in NSCLC.

Study Year	Study type	Sample size	ICI	Antibiotics Used	Outcomes	NCT#	Reference
2023	Interventional	90	Anti–PD-1/PD-L1	No specifics mentioned	Reduced PFS and OS	Not Applicable	(90)
2023	Interventional	115/187	Anti–PD-1/PD-L1	No specifics mentioned	Reduced PFS and OS	NCT04567446	(77)
2020	Interventional	757	Anti-PD-L1	Quinolones, penicillins, and cephalosporins	Reduced OS	NCT01903993/NCT02008227	(91)
2020	Observational	528	Anti-PD-1/PD-L1	Systemic antibiotics	Reduced PFS and OS	Not Applicable	(92)
2018	Retrospective	239	Anti-PD-(L)1/Anti-PD-(L)1+ Anti-CTLA-4	β-lactam and quinolones	Reduced OS	Not Applicable	(93)
2021	Interventional	50	Anti-PD-1/PD-L1	β-lactam and quinolones	Reduced PFS	NCT03563482	(94)
2020	Retrospective	64	Anti-CTLA-4/Anti-PD-1	No specifics mentioned	Reduced PFS and OS	Not Applicable	(95)

However, considering that many patients with NSCLC require ATB treatment over the course of this disease, the current focus of research is to determine what factors can reduce the effects of ATBs on ICI therapy. First, the use of ATBs during the early immunotherapy period may be associated with poor therapeutic outcomes. Interesting data from Zitvogel et al. demonstrated that patients receiving ATBs immediately before or after initiating ICI therapy exhibited poor outcomes (26). Similarly, in a trial involving 72 patients with NSCLC who were treated with nivolumab, the early use of ATBs had also been associated with a shorter OS (98). A meta-analysis conducted on patients with NSCLC established that exposure to ATBs shortly before or after ICI therapy had a notably harmful impact, whereas ATB use later in the disease progression had no significant effect on survival (99). Second, patients with NSCLC that possessed a higher ATB-ICI exposure ratio (defined as “number of days of ATB administration/number of days of ICI administration” for the entire immunotherapy period) also exhibited poor outcomes (100). Third, the effect of ATBs on ICI effectiveness varies depending on the PD-L1 expression levels in patients with advanced NSCLC. Specifically, a deleterious effect has been observed in patients with ≥50% PD-L1 expression but not in those with <50% PD-L1 expression (101). Finally, with all other clinically relevant parameters constant, patients who received a single course of ATBs were observed to exhibit a shorter median OS and PFS, whereas those who received multiple courses or prolonged ATB therapy exhibited the worst overall outcomes (95). Gut microbiota plays a crucial role in immune response. The use of antibiotics affects the efficacy of ICIs by altering the gut microbiota. Alkan et al. showed that the use of antibiotics at the start of ICIS was associated with reduced OS, PFS and objective response rate (ORR) in NSCLC patients (102). This suggests that gut microbiota diversity may be one of the factors predicting ICI efficacy. Therefore, cumulative ATB use is not recommended.

Similar to ATB, PPIs are negative prognostic markers in patients with NSCLC who are undergoing immunotherapy. In a clinical study involving 169 individuals, PPI use was associated with shorter PFS in patients with NSCLC that were treated with atezolizumab (91). Additionally, PPI use has been observed to negatively influence ICI efficacy in patients with NSCLC who received atezolizumab + carboplatin + paclitaxel treatment (ACP) or atezolizumab + bevacizumab + carboplatin + paclitaxel treatment (ABCP) but not in those who received bevacizumab + carboplatin + paclitaxel treatment (BCP) (91). Moreover, a meta-analysis indicated that PPI treatment is associated with a reduced PFS and OS in patients with advanced cancer who were treated with ICIs (103). Thus, when not clearly warranted, PPI medication should be discontinued; additionally, the use of histamine H2-receptor antagonists should be investigated as an alternative to PPI treatment.

In conclusion, there is a consensus on the negative effects of ATBs and PPIs on patients with NSCLC who are receiving immunotherapy. Given that the prohibition of ATB use is impractical for certain patients, modulating ATB-related GM dysbiosis may be a viable strategy to improve clinical outcomes. Therefore, the use of targeted antibiotics that allow immunopotentiating bacteria to proliferate have been recently proposed (104). However, the dosage, frequency, and delivery methods for targeted ATBs remain to be elucidated.

### 4.3 Probiotics, prebiotics, postbiotics, and synbiotics

Recently, probiotics, prebiotics, and postbiotics have gained the attention of clinicians due to their potential to enhance ICI efficacy (**Table 6**). “Probiotic” was first defined in 1965 (105); nonetheless, the most recent definition of probiotic is as follows: “live microorganisms that, when administered in adequate amounts, confer a health benefit on the host” (106). Prebiotics are fibers that are digestible by individual commensal species; these bacteria metabolize the prebiotics, thereby producing secondary metabolites such as SCFAs (107). Any material released or produced via the metabolic activities of microbes that has a direct or indirect positive impact on the host is regarded as a postbiotic (108). Finally, synbiotics are a mix of prebiotics and probiotics and have been proven advantageous in inflammatory bowel disease; however, their impact on cancer is not yet examined in clinical studies (104).

**Table 6:** Clinical trials for the use of prebiotics, probiotics, postbiotics, and synbiotics in combination with ICIs for the treatment of NSCLC.

NCT#	Age	Sample Size	Microbiome−based intervention	Location	Investigator	Status	Study type	Phase	Length
NCT05303493	18+	45	Camu Camu Capsules (*Akkermansia muciniphila*) + ICI	Canada	Bertrand Routy	Not yet recruiting	Interventional	1	04/22–04/27
NCT04699721	18–80	40	*Bifidobacterium trifidum* live powder + Nivolumab + Paclitaxel + Carboplatin AUC5	China	Yang Gao	Recruiting	Interventional	1	07/20–12/27
NCT04105270	18+	82	Restorative microbiota therapy capsules + durvalumab + chemotherapy	United States	Amit Kulkarni	Not yet recruiting	Interventional	2	06/22–01/28
NCT05354102	18+	12	BMC128 (live bio-therapeutic product composed of 4 commensal bacterial strains) + nivolumab	Israel	Ruth Perets	Not yet recruiting	Interventional	1	05/22–05/23
NCT04601402	18+	93	GEN-001 (Each capsule containing ≥ 1×10^11^ CFUs) + Avelumab	United States	Shivaani Kummar	Recruiting	Interventional	1	10/20–01/24
NCT03637803	18+	132	MRx0518 + Pembrolizumab	United States	Shubham Pant	Recruiting	Interventional	1 and 2	01/19–03/24
NCT05094167	18–70	46	Kex02 (*Lactobacillus* and *Bifidobacterium*) + carilizumab + platinum	China	Chunling Jiang	Recruiting	Interventional	Not applicable	10/21–12/23
NCT04909034	20+	30	Ms20+pembrolizumab	Taiwan	Amy Lee	Recruiting	Interventional	2	08/21-06/30

Probiotics, prebiotics, postbiotics, and synbiotics play important roles in assisting immunotherapy in the treatment of NSCLC. A retrospective study involving 294 patients established that probiotic use is associated with favorable clinical outcomes in patients with advanced or recurrent NSCLC who were subjected to anti-PD-1 monotherapy (109). Alternatively, a retrospective study evaluated 118 patients with advanced NSCLC who were treated with ICIs at Kumamoto University Hospital. Significant improvements in PFS and OS were observed in patients who received probiotic *Clostridium butyricum* therapy, even in those who had previously undergone ATB therapy before ICI therapy (110). Castalagin, the active ingredient in camu-camu, can concentrate microorganisms (Ruminococcaceae and *Alistipes*) associated with effective immunotherapeutic responses and can enhance the CD8^+^/FOXP3^+^CD4^+^ ratio in the TME. In preclinical ICI-resistant models, castalagin was determined to re-establish the efficacy of anti–PD-1 in NSCLC (111). Further, in addition to improving survival, 3-IAld (a microbial tryptophan catabolite) has been found to protect mice with ICI-induced colitis from intestinal injury by acting on both the host and microbiota (112). In a direct comparison study, the oral application of a combination of four Clostridiales strains (CC4) was observed to outperform anti-PD-1 therapy in mouse models of NSCLC. Specifically, CC4 therapy has been found to suppress tumor growth by increasing the number of IFN-γ^+^ CD8^+^ T cells and natural killer cells while reducing the number of PD-L1^+^ macrophages (113). A meta-analysis determined that probiotics are favorably associated with the OS and PFS of patients with NSCLC that are being treated with ICIs, but had no effect on ORR (114). Similar results have also been reported in other cancer types. For example, CBM588 appeared to increase the PFS of patients with metastatic RCC who were being treated with nivolumab-ipilimumab (115). Additionally, the adjunctive probiotic *Lactobacillus rhamnosus* probio-M9 was determined to enhance the effect of anti-PD-1 antitumor therapy by repairing antibiotic-disrupted GM in mouse models of colorectal cancer (116). Chen et al. showed that JK5G postbiotics may attenuate immune-related adverse events irAEs and improve quality of life (QoL) and nutritional levels in advanced NSCLC patients receiving ICIs. JK5G postbiotics can also improve gut microbiota structure, increase *Faecalibacterium*, *Ruminococcaceae* relative abundance, fecal butyrate concentration, reduce *Escherichia-Shigella* relative abundance, and improve tumor microenvironment and inflammation (117). A study reveals that probiotic Lactobacillus reuteri (Lr) uses its released dietary tryptophan catabolite indole-3-aldehyde(I3A), it locally promoted the production of interferon-γ by CD8 T cells, thus bolstering ICI (90).

Overall, opinions have been divided regarding the safety of probiotics. Systemic infections, harmful metabolic activities, excessive immunological activation in sensitive individuals, gene transfer, and gastrointestinal side effects are the potential dangers of this therapeutic strategy. On the other hand, critics argue that the overwhelming body of evidence, including a long history of safe probiotic usage and data from clinical trials, alongside animal and *in vitro* research, supports the understanding that probiotics are generally safe for most individuals (118). In general, more advantages than disadvantages have been observed with regard to probiotic treatment. Ultimately, probiotics, prebiotics, postbiotics, and synbiotics are potentially viable strategies as they are less complicated and presumably safer than FMT, which has been associated with increased pathogen transmission.

### 4.4 Bacteriophages

Non-bacterial microorganisms in the GM have received less attention than bacteria, despite their potential importance. Bacteriophages, also known as bacterial viruses or phages, are obligate intracellular parasites that rely on the metabolic apparatus of their bacterial host for multiplication (119). Bacteriophages are the most abundant biological entities on Earth (120). Fluckiger et al. established that *E. hirae* harbors a bacteriophage that can modify immunological responses. After PD-1 inhibition, mice bearing *E. hirae* bacteriophages have been observed to exhibit an increase in the CD8^+^ T lymphocyte response. In patients with lung cancer, the presence of bacteriophages in their feces and the expression of a tape measure protein–cross-reactive antigen in tumors has been linked to better survival following PD-1 treatment (121). Dong et al. screened a strain of selective *Fusobacterium nucleatum*–binding M13 bacteriophages. The introduction of silver nanoparticles onto the surface of the M13 bacteriophages allowed precise scavenging of pro-tumor *F. nucleatum*; consequently, the host’s immune response was enhanced via APC activation and the tumor-immunosuppressive microenvironment was remodeled. Notably, M13-Ag combined with PD1 inhibitors has been observed to significantly prolong OS in a colorectal cancer mouse model (122). Therefore, the use of bacteriophages to modulate the GM for NSCLC immunotherapy is increasingly gaining importance in both basic and translational research.

### 4.5 Diet

Diet plays an essential role in bridging the connection between humans and the microbiota. The GM consumes and utilizes ingested nutrients for basic biological activities; therefore, diet is a pivotal determinant of GM structure and function (123, 124). Recent evidence suggests the use of high-fiber diets for better outcomes in cancer immunotherapy. In a prospective cohort of patients with melanoma that were receiving ICI therapy, intake of a fiber-rich whole-grain diet was strongly correlated with increased PFS. Interestingly, PFS was observed to be the highest in the group that consumed a high-fiber diet but did not consume probiotics. The inhibition of intratumoral IFN-γ T cell responses was assumed to be the cause of the worsened response to immunotherapy observed in those on a low-fiber diet (125). Dietary fermentable fiber content alters the ratio of *Firmicutes* to *Bacteroidetes* in the gut and the lungs. The GM digests fiber, which increases the concentration of circulating SCFAs and influencing the lung immune environment (126). In addition to high-fiber diets, compelling evidence has suggested that fasting-mimicking diets, long-term calorie restrictions, and ketogenic diets may improve anticancer immunosurveillance during immunotherapy (12, 127–131). Overall, several studies have focused on the influence of diet on immunotherapy for NSCLC (**Table 7**).

**Table 7:** Clinical trials on the effects of diet in combination with ICIs for NSCLC treatment.

NCT#	Age	Sample Size	Dietary intervention	Investigator	Status	Study type	Length
NCT04965129	20–90	50	A high protein diet supplemented with fish oil	Wilza AF Peres	Recruiting	Interventional	09/21–12/23
NCT04009122	18+	280	IGEN-0206 (nutritional product)	Carmen Perezagua	Recruiting	Interventional	06/19–12/22
NCT04909034	20+	30	Fermented soybean extract MicrSoy-20	Amy Lee	Recruiting	Interventional	08/21–06/24

## 5 Discussion

Overall, this review provides new insights into the importance of the GM in NSCLC immunotherapy. Wide differences in the diversity and composition of the GM have been discovered between patients with NSCLC who responded and failed to respond to ICI therapy. The bidirectional GLA has recently emerged as a distinct two-way interaction between the lungs and the gut, including both microbial and immunological interactions. Specific GM compositions may communicate with innate and adaptive immune cells, ameliorate ICI responses, and alleviate irAEs in NSCLC. Therefore, numerous studies have explored the optimal bacterial environment within the gut that best enhances immunotherapy effectiveness in NSCLC. FMT, ATBs, PPIs, probiotics, prebiotics, postbiotics, synbiotics, bacteriophages, and diet alterations are among the methods currently being investigated.

To date, four major limitations have been identified regarding the use of the GM as a therapeutic strategy. First, the cause-and-effect relationship between GM and immunotherapy in NSCLC remains unclear. Therefore, larger multicenter collaborations and randomized controlled trials should be performed to identify whether a specific signature GM is a common biomarker for enhancing ICI response and preventing irAEs in NSCLC. Second, it is yet to be clarified whether the current findings on the role of the GM in modulating ICI responses in NSCLC animal models and in patients with other tumor types, such as melanoma or colorectal cancer, also apply to human patients with NSCLC. Third, further investigations into the interaction of GM with other immunotherapies, such as CAR-T cell therapy, dendritic cell vaccines, and adoptive cell transfer, in NSCLC are warranted. Finally, it is important to avoid treating diseases in isolation because organs can interact with each other in various ways. The mechanism of the GLA has primarily been elucidated through the relationship between the GM and asthma, chronic obstructive pulmonary disease, and respiratory infections. Therefore, further research is necessary to understand the effects of the lung microbiota on the gut and the role of the GM in the development and treatment of lung cancer. Specifically, evaluating how the GM modulates NSCLC immunotherapy may further elucidate the implications of GLA interactions in this disease.

Potential risks and side effects of FMT include infection, disease transmission, allergic reactions, etc. Patients who receive FMT may develop intestinal or systemic infections and may develop drug or food allergies. The application of FMT in NSCLC immunotherapy is still in the exploratory stage and has not been widely used at present. Potential risks and side effects of antibiotics include disruption of gut microbiota, development of drug resistance, resulting in intestinal problems such as diarrhea and constipation. Long-term use of antibiotics may also lead to the development of resistance, making antibiotics ineffective. Probiotics may cause drug or food allergies and may interact with antibiotics or other medications. In summary, FMT, antibiotics, and probiotics all carry potential risks and side effects in NSCLC immunotherapy. When using these interventions, it is necessary to weigh the pros and cons according to the specific situation of the patient and choose the most appropriate treatment method. Further studies are needed to determine the long-term efficacy and safety of these interventions (132, 133).

ICI efficacy is often limited by treatment resistance and adverse reactions. Therefore, the development of less invasive biomarkers can identify responders and non-responders early in treatment and significantly improve treatment protocols. Ni et al. found that *Agathbacter*, *Blautia*, *Clostridium* and *Muribaculacea* were more abundant in patients with early NSCLC than in healthy controls. Dysregulation of pathways such as sphingolipid metabolism and sphingolipid signaling pathway may be emerging therapeutic strategies for early NSCLC (134). Zhu et al. showed that the metabolite butyrate of the gut microbiota, promotes the efficacy of anti-PD-1 therapy by modulating T cell receptor (TCR) signaling of cytotoxic CD8 T cells, and the metabolite butyrate may be a very promising therapeutic biomarker for enhancing anti-tumor immunity (135).Sarkar et al. found decreased abundance of *Odoribacter*, *Gordonibacter*, *Stoquefichus*, *Escheria-Shigella*, and *Collinsella* genera and increased abundance of *Clostridium sensu stricto1* in fecal samples of NSCLC patients receiving anti-PD-1 therapy. In contrast, nonresponders to anti-PD-1 immunotherapy showed increased treatment with *Prevotella*, *Porphyromonas*, *Streptococcus*, and *Escherichia-Shigella*,as well as decreased abundance of *Akkermannia*. Gut microbiota have potential utility as a noninvasive biomarker for NSCLC patients to predict response to anti-PD-1 therapy in NSCLC patients and need to be validated in larger prospective studies (136). In the future, it is hoped that gut microbiota and its metabolites will be used as predictive invasiveness biomarkers for response rate to ICI therapy of NSCLC patients, and realize clinical transformation. Therefore, the composition and function of gut microbiota in NSCLC patients should be clarified first, and specific microbial markers related to immunotherapy should be searched. Animal model experiments were conducted to test the effects and side effects of different intervention strategies, and to explore the optimal time and dose of intervention. Finally, testing and validation is performed in clinical trials to determine the optimal treatment regimen. Emerging technologies, such as chip-based gut models, edible capsules for microbiota sampling, and metabolomic analysis may accelerate the development of intervention strategies for gut microbiota.

## 6 Conclusion

Modulation of gut microbiota homeostasis as a new approach to treating NSCLC patients, this approach improves NSCLC therapeutic outcomes through manipulation of gut microbiota, such as probiotics design, FMT, dietary improvement, and modulation of antibiotics application, but all of these approaches lack target specificity. Gut microbiota provides new strategies for the occurrence, development, diagnosis, treatment and prognosis of NSCLC, however, its clinical application still faces the influence of age, gender, disease status and environmental factors on the gut microbiota, which also varies in different individuals, and the immunoregulatory mechanism of gut microbiota is not yet fully understood, which still needs to be extensively validated by a large number of representative preclinical models and clinical trials. In the future, a comprehensive understanding of the mechanisms of probiotic species and gut microbiota in the host and tumor cells is needed, which will help guide the treatment of NSCLC and improve the prognosis.

## Author contributions

HZ: Writing – original draft, Writing – review & editing. ZX: Visualization, Writing – original draft.
